# Supplementary figures and images for: Streptococcus pyogenes Forms Serotype- and Local Environment-Dependent Interspecies Protein Complexes
Source: mSystems. 2021 Sep 28;6(5):e00271-21. doi: 10.1128/mSystems.00271-21 (PMC8547449; doi:10.1128/mSystems.00271-21)

A

## M28-IgA

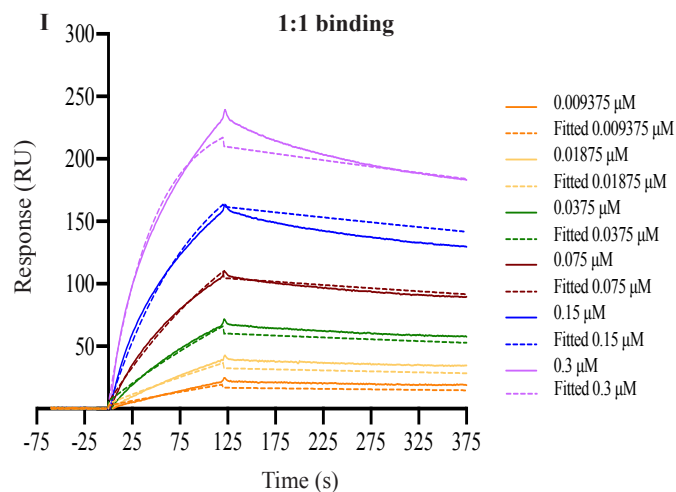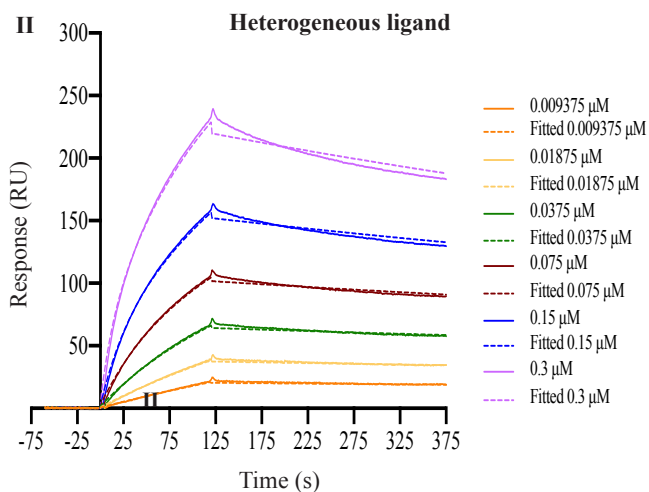

B

## M1-IgA

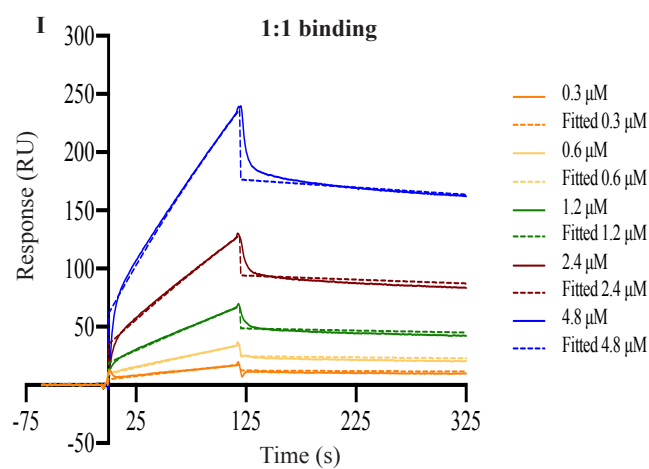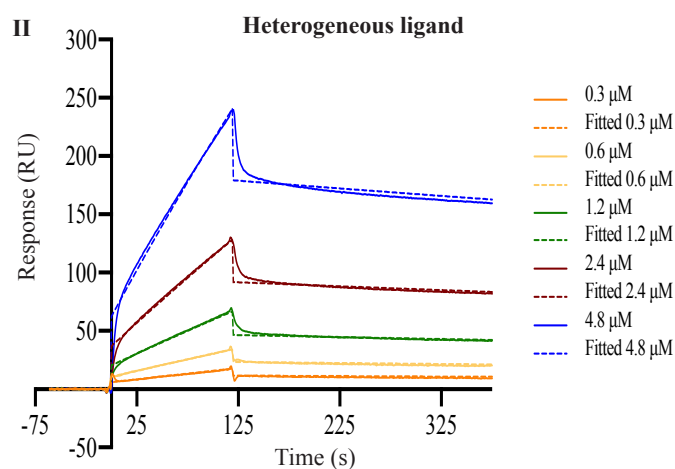

C

## M28-C4BP

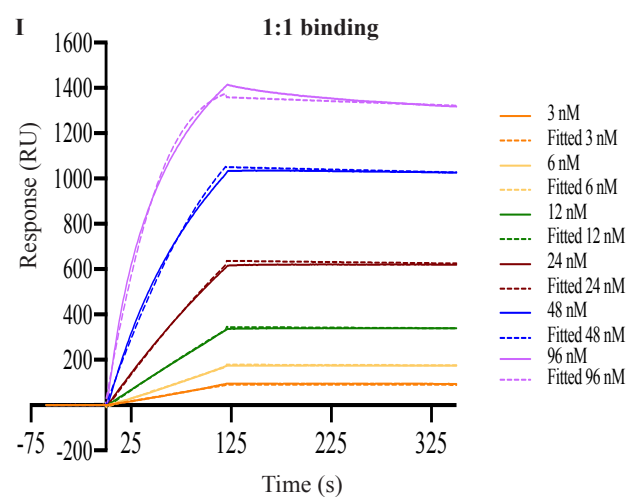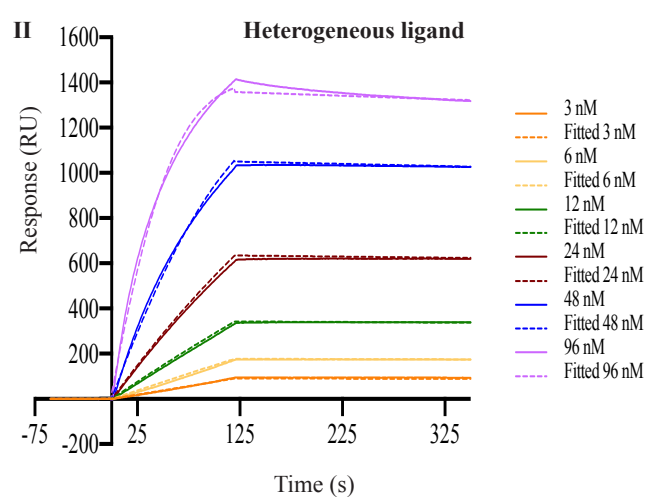

D

| Interaction | 1-1 binding | Heterogeneous ligand |
|-------------|-------------|----------------------|
| M28-IgA     | 25.6        | 6.22                 |
| M1-IgA      | 0.275       | 0.270                |
| M28-C4BP    | 88.1        | 98.9                 |

Figure-S3

Supplement: FIG S3 [file msystems.00271-21-sf003.pdf]

**A**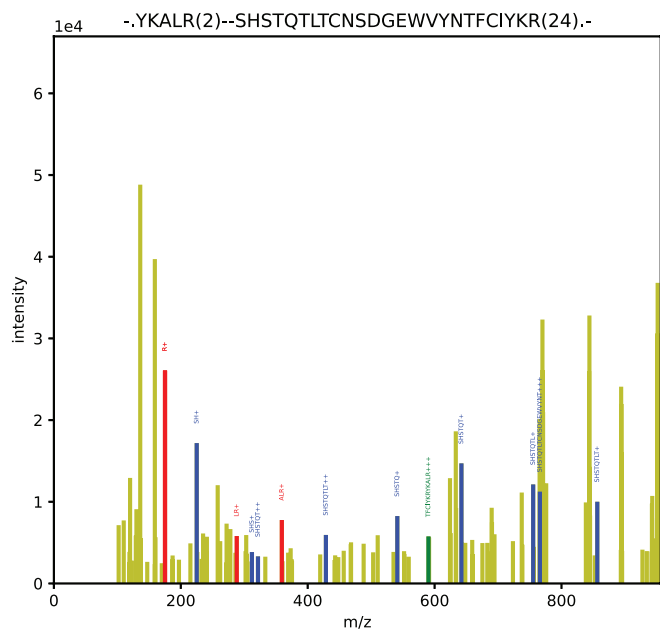**B**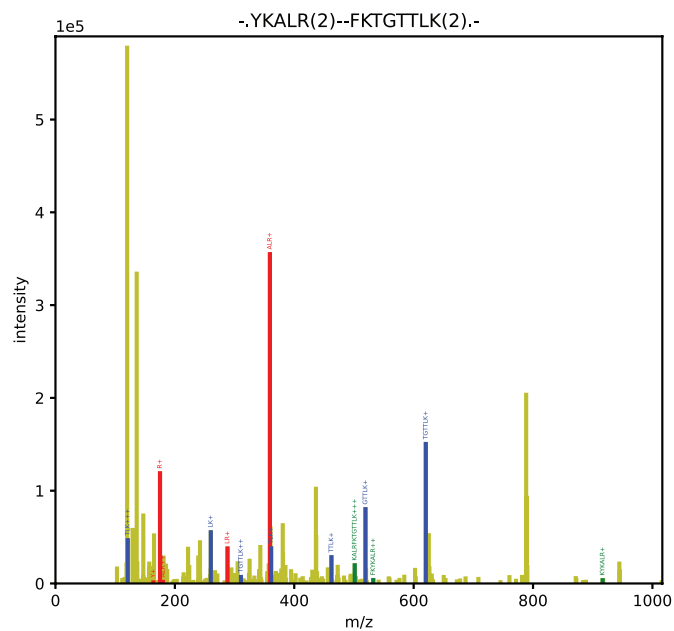**C**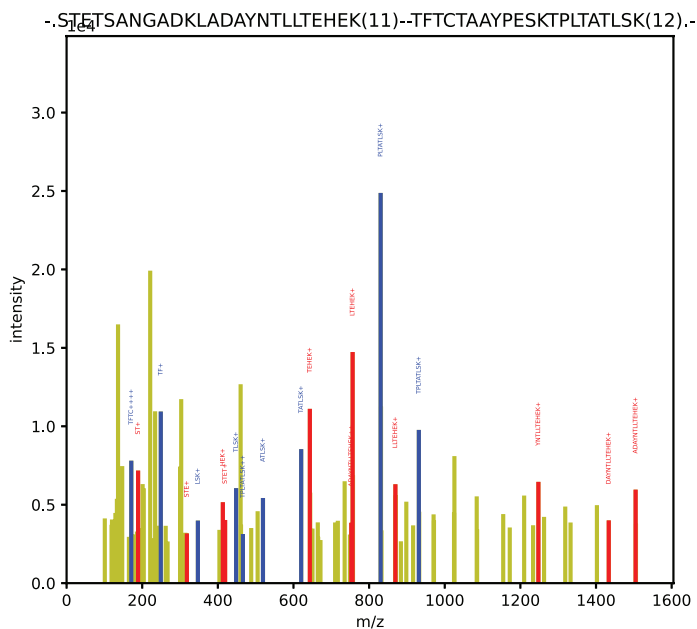**D**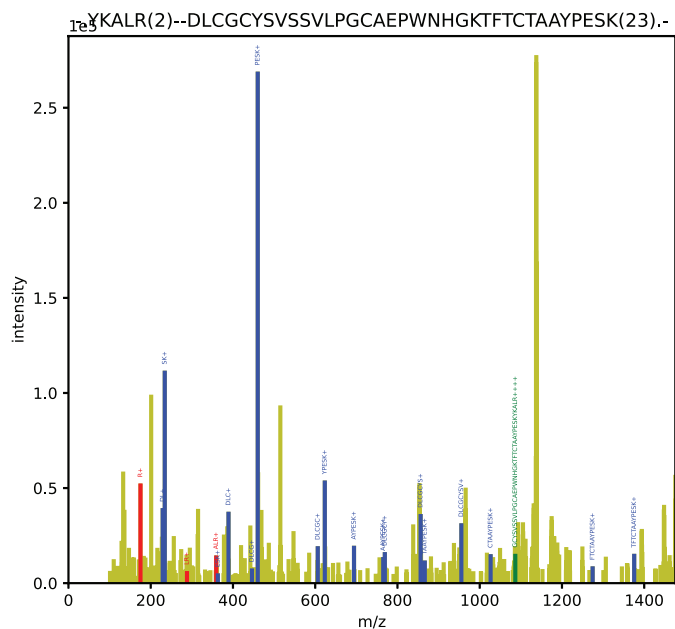**E**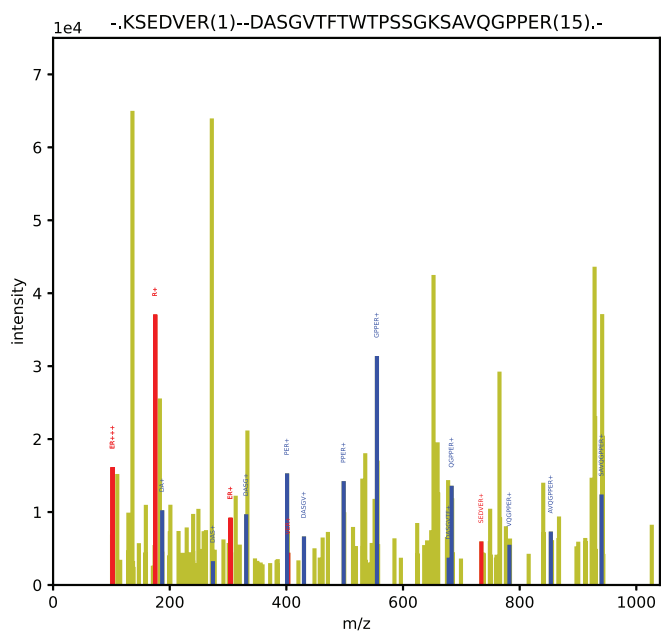**F**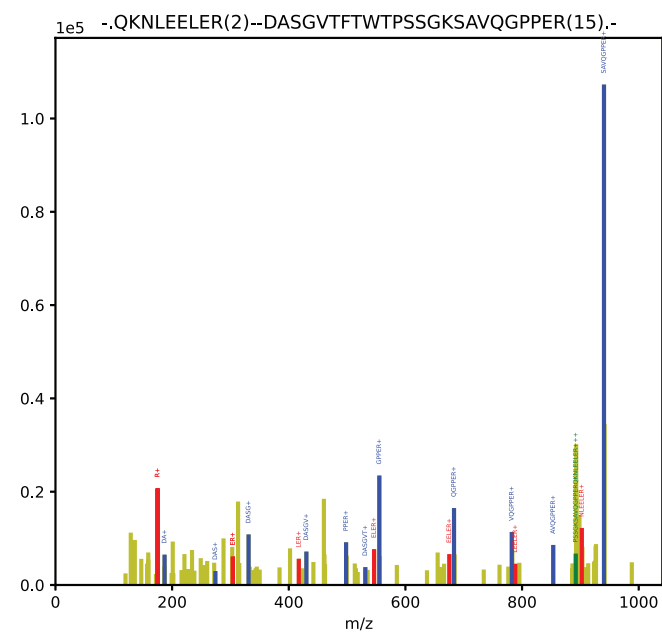**Figure-S4**

# I

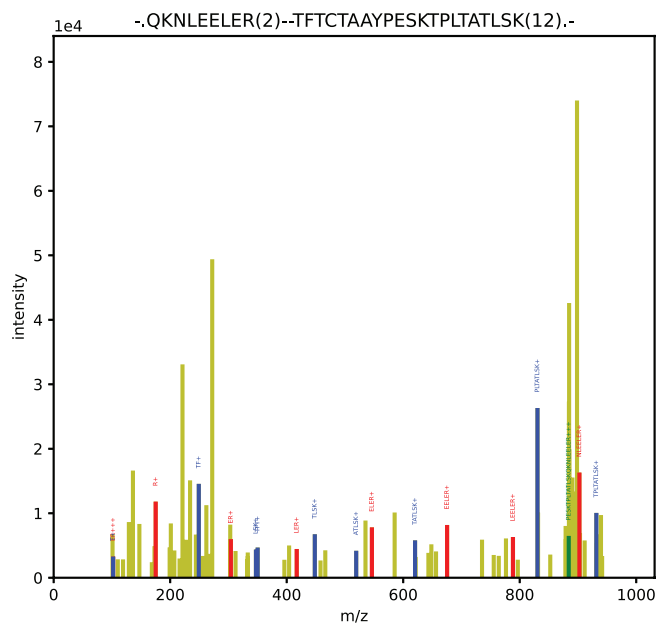

# J

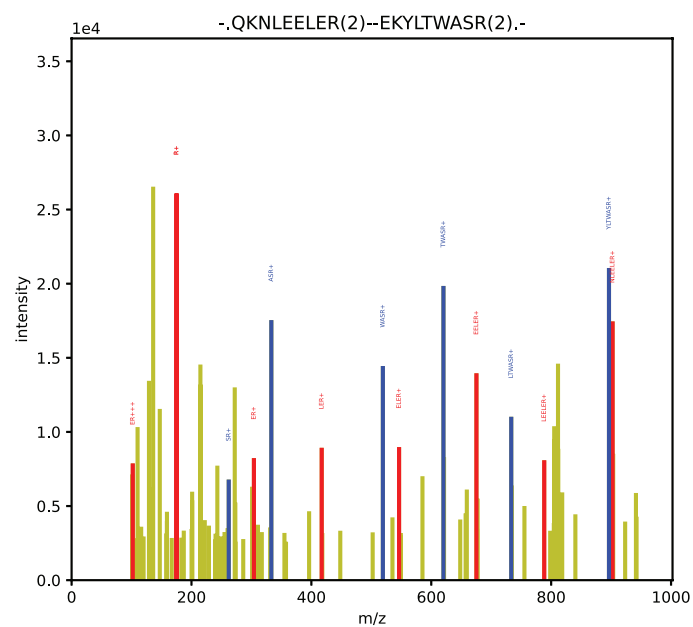

# I

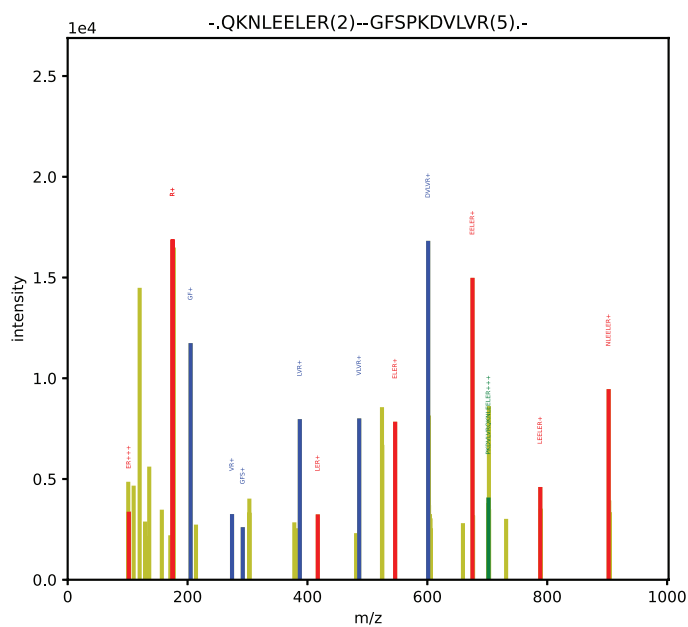

# J

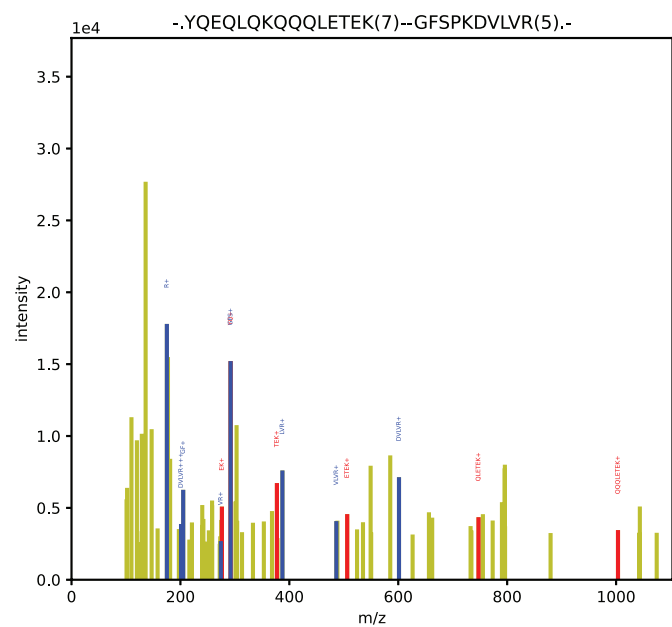

## K

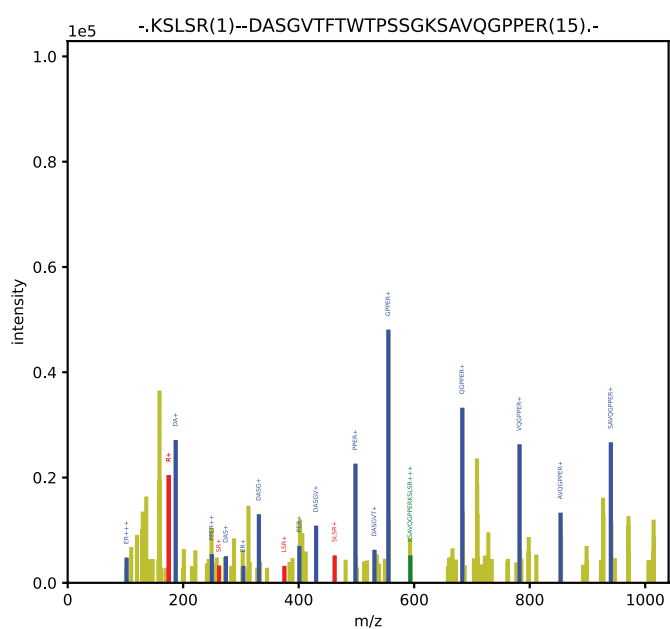

L

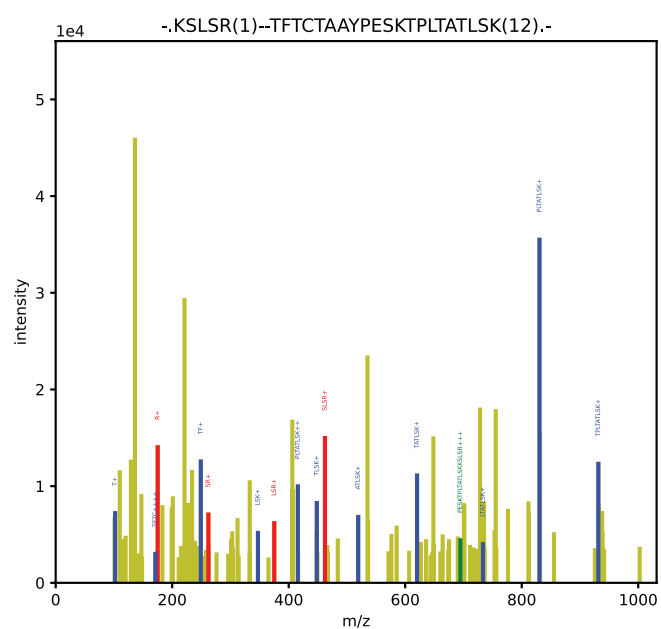

### Figure-S4

Supplement: FIG S4 [file msystems.00271-21-sf004.pdf]
