# Supplementary material for: Streptococcus pyogenes Forms Serotype- and Local Environment-Dependent Interspecies Protein Complexes
Source: mSystems. 2021 Sep 28;6(5):e00271-21. doi: 10.1128/mSystems.00271-21 (PMC8547449; doi:10.1128/mSystems.00271-21)
Supplement: TABLE S1 [file msystems.00271-21-st001.docx]

**Supplementary table-1 (ST1)**

| **M proteins** | **Amino acid sequences** |
| --- | --- |
| M1(Q99XV0) | **M**NGDGNPREVIEDLAANNPAIQNIRLRHENKDLKARLENAMEVAGRDFKRAEELEKAKQALEDQRKDLETKLKELQQDYDLAKESTSWDRQRLEKELEEKKEALELAIDQASRDYHRATALEKELEEKKKALELAIDQASQDYNRANVLEKELETITREQEINRNLLGNAKLELDQLSSEKEQLTIEKAKLEEEKQISDASRQSLRRDLDASREAKKQVEKDLANLTAELDKVKEDKQISDASRQGLRRDLDASREAKKQVEKDLANLTAELDKVKEEKQISDASRQGLRRDLDASREAKKQVEKALEEANSKLAALEKLNKELEESKKLTEKEKAELQAKLEAEAKALKEQLAKQAEELAKLRAGKASDSQTPDTKPGNKAVPGKGQAPQAGTKPNQNKAPMKETKRENLYFQSWSHPQFEKYPYDVPDYAHHHHHH |
| M3(W0T370) | MDARSVNGEFPRHVKLKNEIENLLDQVTQLYTKHNSNYQQYNAQAGRLDLRQKAEYLKGLNDWAERLLQELNGEDVKKVLGKVAFEKDDLEKEVKELKEKIDKKEKEYQDLDKDFDLAKQGYVLSDKRHQQELEEKEKKVTEATAKVGQISEELETVKQKVESTMQDLTEKQNRVSQLEQE  LATTKQNAKEDFELAALANAADKQKLEAKIADLETKLKEAKEDFELAALGHQHAHNEYQAKLAEKDDQIKQLEEQKQILDASRKGTARDLEAVRQAKKATEAELNNLKAELAKVTEQKQILDASRKGTARDLEAVRQAKAQVEAALKQLEEQNRISEASRKGLRRDLDASREAKKQVEKDLANLTAELDKVKEEKQISDASRQGLRRDLDASREAKKQVEKALEEANSKLAALEKLNKELEESKKLTEKEKAELQAKLEAEAKALKEQLAKQAEELAKLRAGKASDSQIPDTKPGNKAVPGKGQAPQAGTKPNQNKAPMKETKRENLYFQSWSHPQFEKYPYDVPDYAHHHHHH |
| M5(P02977) | MAVTRGTINDPQRAKEALDKYELENHDLKTKNEGLKTENEGLKTENEGLKTENEGLKTEKKEHEAENDKLKQQRDTLSTQKETLEREVQNTQYNNETLKIKNGDLTKELNKTRQELANKQQESKENEKALNELLEKTVKDKIAKEQENKETIGTLKKILDETVKDKIAKEQENKETIGTLKKILDETVKDKLAKEQKSKQNIGALKQELAKKDEANKISDASRKGLRRDLDASREAKKQLEAEHQKLEEQNKISEASRKGLRRDLDASREAKKQLEAEHQKLEEQNKISEASRKGLRRDLDASREAKKQVEKALEEANSKLAALEKLNKELEESKKLTEKEKAELQAKLEAEAKALKEQLAKQAEELAKLRAGKASDSQTPDTKPGNKAVPGKGQAPQAGTKPNQNKAPMKETKRENLYFQSWSHPQFEKYPYDVPDYAHHHHHH |
| M28(W0T1Y4) | MAESPKSTETSANGADKLADAYNTLLTEHEKLRDEYYTLIDAKEEEPRYKALRGENQDLREKEGKYQDKIKKLEEKEKNLEKKSEDVERHYLKKLDQEHKEQEERQKNLEELERQSQREIDKRYQEQLQKQQQLETEKQISEASRKSLSRDLEASRAAKKDLEAEHQKLKEEKQISDASRQGLSRDLEASRAAKKDLEAEHQKLKEEKQISDASRQGLSRDLEASREAKKKVEADLAEANSKLQALEKLNKELEEGKKLSEKEKAELQARLEAEAKALKEQLAKQAEELAKLKGNQTPNAKVAPQANRSRSAMTQQKRENLYFQSWSHPQFEKYPYDVPDYAHHHHHH |
| TEV cleaved M28(W0T1Y4) | MAESPKSTETSANGADKLADAYNTLLTEHEKLRDEYYTLIDAKEEEPRYKALRGENQDLREKEGKYQDKIKKLEEKEKNLEKKSEDVERHYLKKLDQEHKEQEERQKNLEELERQSQREIDKRYQEQLQKQQQLETEKQISEASRKSLSRDLEASRAAKKDLEAEHQKLKEEKQISDASRQGLSRDLEASRAAKKDLEAEHQKLKEEKQISDASRQGLSRDLEASREAKKKVEADLAEANSKLQALEKLNKELEEGKKLSEKEKAELQARLEAEAKALKEQLAKQAEELAKLKGNQTPNAKVAPQANRSRSAMTQQKRENLYFQ |
| M49(P16947) | MAEKKVEAKVEVAENNVSSVARREKELYDQIADLTDKNGEYLERIGELEERQKNLEKLEHQSQVAADKHYQEQAKKHQEYKQEQEERQKNQEQLERKYQREVEKRYQEQLQKQQQLETEKQISEASRKSLSRDLEASREAKKKVEADLAALTAEHQKLKEEKQISDASRQGLSRDLEASREAKKKVEADLAALTAEHQKLKEEKQISDASRQGLSRDLEASREAKKKVEADLAEANSKLQALEKLNKELEEGKKLSEKEKAELQARLEAEAKALKEQLAKQAEELAKLKGNQTPNAKVAPQANRSRSAMTQQKRENLYFQSWSHPQFEKYPYDVPDYAHHHHHH |
| M89(W0T3V8) | MDSDNINRSVSVKDNEKELHNKIADLEEERGEHLDKIDELKEELKAKEKSSENVERHYLRKLDQEYKEQQERQKNLEELERQSQREVEKRYQEQLQKQQQLETEKQISEASRKSLSRDLEASRAAKKDLEAEHQKLKEEKQISDASRQGLSRDLEASRAAKKDLEAEHQKLKEEKQISDASRQGLSRDLEASRAAKKDLEAEHQKLKEEKQISDASRQGLSRDLEASREAKKKVEADLAEANSKLQALEKLNKELEEGKKLSEKEKAELQAKLEAEAKALKEQLAKQAEELAKLKGNQTPNAKVAPQANRSRSAMTQQKRENLYFQSWSHPQFEKYPYDVPDYAHHHHHH |
