# Supplementary material for: Streptococcus pyogenes Forms Serotype- and Local Environment-Dependent Interspecies Protein Complexes
Source: mSystems. 2021 Sep 28;6(5):e00271-21. doi: 10.1128/mSystems.00271-21 (PMC8547449; doi:10.1128/mSystems.00271-21)
Supplement: TABLE S2 [file msystems.00271-21-st002.docx]

**Supplementary table-2 (ST2)**

| **Peptide 1** | **Protein 1** | **Peptide 2** | **Protein 2** | **Crosslink corresponding to spectrum (Fig S4)** |
| --- | --- | --- | --- | --- |
| Y**K**ALR | M28 | SHSTQTLTCNSDGEWVYNTFCIY**K**R | C4BP | A |
| Y**K**ALR | M28 | F**K**TGTTLK | C4BP | B |
| STETSANGAD**K**LADAYNTLLTEHEK | M28 | TFTCTAAYPES**K**TPLTATLSK | IgA-Fc | C |
| Y**K**ALR | M28 | DLC  GCYSVSSVLPGCAEPWNHG**K**TFTCTAAYPESK | IgA-Fc | D |
| **K**SEDVER | M28 | DASGVTFTWTPSSG**K**SAVQGPPER | IgA-Fc | E |
| Q**K**NLEELER | M28 | DASGVTFTWTPSSG**K**SAVQGPPER | IgA-Fc | F |
| Q**K**NLEELER | M28 | TFTCTAAYPES**K**TPLTATLSK | IgA-Fc | G |
| Q**K**NLEELER | M28 | E**K**YLTWASR | IgA-Fc | H |
| Q**K**NLEELER | M28 | GFSP**K**DVLVR | IgA-Fc | I |
| YQEQLQ**K**QQQLETEK | M28 | GFSP**K**DVLVR | IgA-Fc | J |
| **K**SLSR | M28 | DASGVTFTWTPSSG**K**SAVQGPPER | IgA-Fc | K |
| **K**SLSR | M28 | TFTCTAAYPES**K**TPLTATLSK | IgA-Fc | L |
